# Dyspnea in patients with atrial fibrillation: Mechanisms, assessment and an interdisciplinary and integrated care approach

**DOI:** 10.1016/j.ijcha.2022.101086

**Published:** 2022-07-19

**Authors:** Rachel M.J. van der Velden, Astrid N.L. Hermans, Nikki A.H.A. Pluymaekers, Monika Gawalko, Adrian Elliott, Jeroen M. Hendriks, Frits M.E. Franssen, Annelies M. Slats, Vanessa P.M. van Empel, Isabelle C. Van Gelder, Dick H.J. Thijssen, Thijs M.H. Eijsvogels, Carsten Leue, Harry J.G.M. Crijns, Dominik Linz, Sami O. Simons

**Affiliations:** a Department of Cardiology, Maastricht University Medical Centre and Cardiovascular Research Institute Maastricht, Maastricht, the Netherlands; b Institute of Pharmacology, West German Heart and Vascular Centre, University Duisburg-Essen, Germany; c 1st Department of Cardiology, Doctoral School, Medical University of Warsaw, Warsaw, Poland; d Centre for Heart Rhythm Disorders, University of Adelaide and Royal Adelaide Hospital, Adelaide, Australia; e Caring Futures Institute, College of Nursing and Health Sciences, Flinders University, Adelaide, Australia; f Department of Research and Development, Ciro, Horn, the Netherlands; g NUTRIM School of Nutrition and Translational Research in Metabolism, Maastricht University, Maastricht, the Netherlands; h Department of Respiratory Medicine, Maastricht University Medical Centre (MUMC+), Maastricht, the Netherlands; i Department of Respiratory Medicine, Leiden University Medical Centre (LUMC), Leiden, the Netherlands; j Department of Cardiology, University of Groningen, University Medical Center Groningen, Groningen, the Netherlands; k Department of Physiology, Radboud Institute for Health Sciences, Radboud University Medical Centre, Nijmegen, the Netherlands; l Department of Psychiatry and Psychology, Maastricht University Medical Centre (MUMC+), Maastricht, the Netherlands; m School of Mental Health and Neuroscience (MHeNS), Maastricht University, Maastricht, the Netherlands; n Department of Cardiology, Radboud University Medical Centre, Nijmegen, the Netherlands; o Department of Biomedical Sciences, Faculty of Health and Medical Sciences, University of Copenhagen, Copenhagen, Denmark

**Keywords:** Atrial fibrillation, Dyspnea, Exercise intolerance, Symptom assessment, Comorbidities, Mechanisms

## Abstract

Atrial fibrillation (AF) is the most common sustained heart rhythm disorder and is often associated with symptoms that can significantly impact quality of life and daily functioning. Palpitations are the cardinal symptom of AF and many AF therapies are targeted towards relieving this symptom. However, up to two-third of patients also complain of dyspnea as a predominant self-reported symptom. In clinical practice it is often challenging to ascertain whether dyspnea represents an AF-related symptom or a symptom of concomitant cardiovascular and non-cardiovascular comorbidities, since common AF comorbidities such as heart failure and chronic obstructive pulmonary disease share similar symptoms. In addition, therapeutic approaches specifically targeting dyspnea have not been well validated. Thus, assessing and treating dyspnea can be difficult.

This review describes the latest knowledge on the burden and pathophysiology of dyspnea in AF patients. We discuss the role of heart rhythm control interventions as well as the management of AF risk factors and comorbidities with the goal to achieve maximal relief of dyspnea. Given the different and often complex mechanistic pathways leading to dyspnea, dyspneic AF patients will likely profit from an integrated multidisciplinary approach to tackle all factors and mechanisms involved. Therefore, we propose an interdisciplinary and integrated care pathway for the work-up of dyspnea in AF patients.

## 1 Introduction

Atrial fibrillation (AF) is the most common sustained cardiac arrhythmia, with a prevalence of up to 4% in the general population [1]. Palpitations are the cardinal symptom of AF [2]. Current treatment strategies are targeted at controlling such symptoms and include either rate control, for instance beta blockers, or rhythm control treatment such as ablation. These therapies are successful in treating patient symptoms [3], [4].

Dyspnea is another common presenting symptom of AF. Studies have shown that it can be the presenting symptom in up to two-thirds of patients **(**Table 1**)**. This symptom is more common when AF is accompanied by a comorbid disease, such as heart failure (HF) or chronic obstructive pulmonary disease (COPD) [5]. The connection between dyspnea and underlying comorbidities makes it challenging for physicians to appraise and treat dyspnea adequately in the setting of AF, since it may be difficult to ascertain whether dyspnea represents an AF-related symptom or a symptom of a cardiovascular or non-cardiovascular comorbidity. Additionally, the intrinsically subjective nature of dyspnea complicates proper evaluation. Indeed, the best way to approach dyspnea in a (multimorbid) AF patient is unknown.

**Table 1:** Prevalence of dyspnea in patients with atrial fibrillation.

**Study**	**Country**	**Number of participants**	**Mean age**	**Atrial fibrillation type**				**Heart failure (%)**	**Obstructive respiratory diseases (%)**	**Dyspnea most common symptom**	**Prevalence of dyspnea (%)**	**Symptom assessment**
				First episode (%)	Paroxysmal (%)	Persistent (%)	Permanent (%)					
Bin Salih et al, 2011^S1^	Saudi Arabia	720	unknown	-	21.8	78.1^a^	-	26.2	31.8	Yes	59.3	Self-reported symptoms
Blum et al, 2017^S2^	Switzerland	1542	70.4 ± 10.7 67.2 ± 11.9^b^	–	55.7	23.9	20.4	20.4	–	Yes^c^	25.9	Questionnaires by study personnel, unspecified
Dhungel et al, 2017^S3^	Nepal	205	63.95 ± 16.5	–	43.4	36.1	20.5	56.5	12.3	Yes	41	Self-reported symptoms
Freestone et al, 2003^S4^	Malaysia	40	65 ± 10.3	52.5	17.5	30^a^	–	40	7.5	Yes	40	Self-reported symptoms
Guerra et al, 2017^S5^	Multiple countries in Europe	3607	66 ± 12.6	17.6	28.2	22.8	29.4	28.7	12.0	No	42.6	Self-reported symptoms
Lip et al, 2015^S6^	Multiple countries in Europe	3119	68.8 ± 11.5	30.3	26.5	26	17.3	47.5	11.0	No	53.7	Unspecified
Lok et al, 1995^S7^	Hong Kong	291	73 ± 12	–	–	–	–	22	9.6	No	38.1	Self-reported symptoms
Schnabel et al, 2018^S8^	Multiple countries in Europe	6196	71.8 ± 10.4	–	–	–	–	28.6	–	No	66.2	EHRA score

Abbreviations: *EHRA = European Heart Rhythm Association*.

NB: references from this table can be found in the supplementary material.

a persistent and permanent together defined as ‘’chronic’’ ^b^mean age in women and men, respectively ^c^In patients with non-paroxysmal AF.

The aim of this review is to provide an integral overview of dyspnea in AF. We will discuss the pathophysiology of dyspnea in AF and describe the important role of comorbidities. Next, dyspnea assessment and the efficacy of current AF therapy to provide dyspnea relief is presented and the need for a multidisciplinary approach is highlighted. Since dyspnea and exercise intolerance are closely linked [6], we will use both interchangeably throughout this review.

## 2 Pathophysiological mechanisms of dyspnea related to AF episodes

**Fig. 1:**
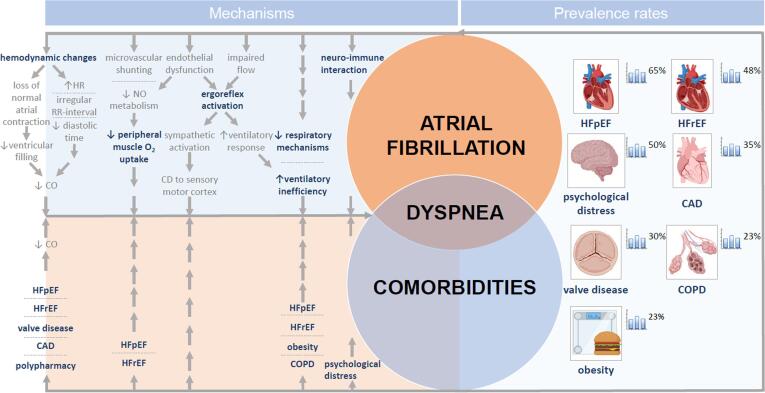
**Mechanisms of dyspnea and prevalence rates of common dyspnea-related comorbidities and risk factors in atrial fibrillation patients.** Abbreviations: CAD = coronary artery disease, CD = corollary discharge, CO = cardiac output, COPD = chronic obstructive pulmonary disease, HFpEF = heart failure with preserved ejection fraction, HFrEF = heart failure with reduced ejection fraction, HR = heart rate, NO = nitric oxide. *Composed of anxiety, depression and symptom preoccupation.

Several mechanisms have been proposed that may play a role in the pathophysiology and perception of dyspnea in AF **(**Fig. 1**)**.

### 2.1 Central hemodynamic aspects

Intuitive mechanisms for dyspnea and reduced exercise performance in AF are linked to impairments in hemodynamic responses to physical activity. First, the loss of a normal atrial contraction as a result of AF leads to reduced ventricular filling and subsequently reduced cardiac output [7]. Second, cardiac output may be further impaired in AF due to an increased ventricular rate, an irregular RR-interval, atrioventricular valve regurgitation, and reduced diastolic time or by a detrimental effect of ischemia [8]. HF has significant impact on exercise tolerance in AF [9]. In HF with reduced ejection fraction (HFrEF) there is an increased heart rate response during exercise in AF compared to HFrEF patients in sinus rhythm, reflecting increased sympathetic activity in AF [10]. Stroke volume during exercise is similarly reduced in HFrEF and AF leading to a compromised exercise performance which is around 20% lower than in sinus rhythm [11]. Several studies on invasive hemodynamic monitoring have shown that cardiac output during exercise increases after conversion to sinus rhythm [12]. Moreover, conversion to sinus rhythm improved maximum oxygen consumption, irrespective of underlying heart disease, and significantly improved symptoms in terms of New York Heart Association class [13].

Heart failure with preserved ejection fraction (HFpEF) also impairs exercise tolerance. Exercise intolerance in AF is strongly associated with elevated left ventricular filling pressure [14]. This sometimes occurs only during exercise, suggesting early-stage HFpEF [15]. Exercised-induced elevated left ventricular filling pressures are relatively frequent in AF [16]. In HFpEF left ventricular filling is more dependent on left atrial contraction and subsequently AF is poorly tolerated [15].

### 2.2 Peripheral muscle oxygen uptake

Additionally, peripheral muscle oxygen uptake may play a role in the pathophysiology and perception of dyspnea in AF. Oxygen transport (VO2) can be described by the product of cardiac output and arterial-venous O2 content difference. Previous studies have shown that blood flow to the legs remains preserved even in severe HF suggesting a peripheral limitation to exercise in these patient [17]. Invasive exercise studies in HFrEF and HFpEF have indeed confirmed that besides a lower convective component (i.e., low cardiac output) a peripheral muscle diffusion disorder also contributes to the exercise intolerance [18], [19]. Impaired nitric oxide metabolism and microvascular shunting in the muscles are putative mechanisms [20]. In AF, these mechanisms can be exaggerated further impairing the convective component of oxygen transport to the peripheral muscle [21]. Additionally, AF is associated with endothelial dysfunction, leading to a low bioavailability of nitric oxide [21].

### 2.3 Neural involvement (reflexes)

Both the impaired flow and endothelial dysfunction can lead to increased exercise pressor reflex (EPR) activity in AF [10], [22]. In healthy subjects afferent signals from the contracting muscles reflexively activate the sympathetic nerve system; this is called the EPR [23]. The increased sympathetic nerve system activity in AF leads to an exaggerated response of the sympathetic nerve system after stimulating the EPR [10]. This subsequently leads to an augmented tachypneic ventilatory response to exercise such as is seen in HF. The mismatch between the anticipated corollary discharge to the somatosensory cortex and the increased afferent feedback signals may give rise to a feeling of increased effort and leg fatigue [24]. Restoration to sinus rhythm leads to a decrease in the EPR [25] and improves microcirculation [26].

### 2.4 Respiratory mechanisms and ventilatory inefficiency

Healthy individuals rarely have a ventilatory limitation to exercise [27]. Exercise can be limited by ventilation if lung function is impaired, such as seen in COPD [28]. Likewise, increased ventilatory demands, lower lung compliance and excessive loading can give rise to ventilatory limitations such as seen in HF and (morbid) obesity [24]. It can be assumed that respiratory mechanics may also contribute to the exercise limitation in an AF patient with multiple comorbidities. Moreover, AF itself may be linked to an increased ventilatory inefficiency, as depicted by a steeper minute ventilation/carbon dioxide production slope during exercise (Ve slope). This can either be caused by the autonomic dysfunction in AF and the subsequent hyperpneic response after EPR stimulation, but ventilatory efficiency is also closely linked to right ventricular function and pulmonary vascular tone [29]. AF-associated backward failure and subsequent fluid accumulation may therefore worsen ventilatory efficiency such as seen in HFpEF.

### 2.5 Impact of negative affectivity

The emotional state of a person also modulates dyspnea. Anxiety, anger and depression can increase symptoms of dyspnea out of proportion with regard to the cardiorespiratory dysfunction [30]. In AF, psychological distress is common and it is associated with an increased symptom perception [31]. The exact cause-effect relationship between affective disorders and AF can be difficult to disentangle given that stress-related psychological conditions do not only increase symptom perception but may also directly impact AF. It is postulated that low-grade chronic inflammation, as seen in AF, can sensitize the cortical-amygdala circuitry to overshoot during threat responses [32]. This activates the neuro-immune network and induces autonomous hyperreactivity [32]. The autonomic hyperreactivity can contribute to the peripheral limitation as well as the ventilatory inefficiency during exercise seen in AF. Negative emotions therefore do not only increase dyspnea perception in AF but also have a direct link to exercise intolerance.

## 3 The importance of comorbidities for dyspnea in AF patients

Comorbidities are common in AF and their relevance increases in patients with dyspnea, because many of these comorbidities affect the success of AF management [33]. Fig. 1 gives an overview of the prevalence rates of the most common dyspnea-related comorbidities and risk factors in AF patients.

*Cardiovascular disease.* HF is highly prevalent among AF patients (HF in general 20–48%, with equal distribution of HFrEF and HFpEF) [34], [35]. However, due to a change in definition and a rising prevalence, recently even higher prevalence rates of HFpEF in AF patients have been reported (65%) [36] and especially in AF patients with dyspnea, the prevalence of occult HFpEF seems extremely high (91–98%) [37]. The presence of HF in AF patients appeared to be strongly associated with more symptoms and less frequently controlled AF [35]. Other cardiovascular comorbidities such as valvular heart disease or coronary artery disease (CAD) are also common in patients with AF, with prevalence rates of 30% and 18–46%, respectively [38], [39]. Since dyspnea may be the only presenting symptom of CAD, this is an important condition to consider in AF patients with dyspnea. Although uncommon, pulmonary hypertension, e.g. caused by chronic pulmonary embolisms (chronic thromboembolic pulmonary hypertension) is an important comorbidity with dyspnea usually being the first presenting symptom.

*Non-cardiovascular comorbidities.* Besides cardiovascular comorbidities, several non-cardiovascular comorbidities contribute to dyspnea. COPD is present in up to 23% of AF patients [28], [40]. Dyspnea is the hallmark symptom of COPD and as such it is an important comorbidity to exclude in patients with AF presenting with dyspnea. Diagnosing COPD has important consequences since it negatively affects quality of life and symptoms in AF patients and is associated with lower success rates of catheter ablation [28], [41]. In addition, obesity is a known cause of dyspnea. Approximately 23% of AF patients are obese and obesity negatively impacts AF burden and AF symptom burden [42], [43]. Furthermore, the prevalence of psychological distress, including anxiety, depression and symptom preoccupation in patients with AF is high (25–50%) [31], [44]. Screening for depression and anxiety seems a reasonable step in AF patients because the presence of psychological distress may negatively affect treatment outcome of heart rhythm interventions [45], [46].

In conclusion the clinical relevance of comorbidities is higher in patients with AF and dyspnea. Since comorbidities are highly prevalent in dyspneic AF patients, dyspnea could be used as a diagnostic clue to systematically search for concomitant conditions in AF patients, since assessment of comorbidities is crucial to adopt the most ideal treatment strategy. In addition, an accurate evaluation of symptom burden, symptom-rhythm correlation and effect on the functional status of AF patients is also of importance.

## 4 Dyspnea assessment

There is a need for adequate assessment tools for dyspnea, since dyspnea assessment in daily clinical practice is usually subjective and self-reported. The most frequently used classification scales, the European Heart Rhythm Association (EHRA) classification [47] and the Canadian Cardiovascular Society Severity of Atrial Fibrillation scale [48] do not specifically address dyspnea in their score **(Supplement** Table 1). Importantly, both scales assess symptoms during an AF episode, possibly underestimating the illness burden in paroxysmal AF in the absence of such episodes. Numerous other questionnaires are currently used for AF research purposes only **(**Supplement Table 1) but these questionnaires were not specifically designed to assess dyspnea as a main symptom or address all the different domains of dyspnea [49]. In addition, the assessment of a symptom-rhythm correlation is an important step in assessing dyspnea, since a clear symptom-rhythm correlation between reported symptoms and actual documented AF episodes is only present in half of the cases [50]. However, this is not currently incorporated into the standard assessment of dyspnea in AF patients.

Burden of dyspnea should not only include severity but should also assess time spend with dyspnea, which requires gathering information on both severity and frequency preferably by a prospective repetitive longitudinal momentary assessment. Future questionnaires should therefore include dyspnea specific (sub)scales that are easy to apply, provide sensitive and valid information regarding all domains of dyspnea and that are responsive to intervention, or dyspnea should be systematically assessed by momentary assessment tools (e.g., through mobile health applications)[51]. In **Supplement Table 2**, we propose some requirements for dyspnea measurement in AF patients which could be incorporated in such a mobile health tool.

## 5 Managing dyspnea in AF

Current guidelines recommend the simple Atrial fibrillation Better Care holistic pathway, which includes both symptom management and comorbidity optimization next to anticoagulation therapy [33].

### 5.1 Symptom management

Importantly, not all dyspnea is always directly related to AF episodes [52]. In patients with paroxysmal AF, the temporal relationship between AF paroxysms can be interrogated during history taking. In patients with persistent AF, cardioversion may be used to restore sinus rhythm and test for dyspnea relief to assess symptom-rhythm correlation. However, the current approach of symptom assessment around rhythm control by electrical cardioversion (ECV), once before ECV and once within 1-month follow-up, may be suboptimal and rarely identified a symptom-rhythm correlation [53]. Although studies on dyspnea improvement with rate or rhythm control strategies are scarce, rhythm control by catheter ablation has been shown to reduce dyspnea at rest and on exertion, which is in line with the overall beneficial effect of catheter ablation on arrhythmia-related symptoms [54].

### 5.2 Cardiovascular and comorbidity optimization

There is some evidence that targeting cardiovascular risk factors and comorbidities also provides dyspnea relief in patients with AF. De With et al.[55], showed that optimizing blood pressure, improving HF treatment and improving the overall cardiovascular risk profile improves quality of life and dyspnea in patients with both AF and HF. Several observational trials [56–58] similarly showed that AF symptom burden and symptom severity scores declined significantly more with aggressive risk factor management and weight reduction in those with obesity. The greater the reduction in weight, the more effect on AF symptoms was achieved [56].

In addition, increasing number of studies reveal a central role for regular exercise in the primary and secondary prevention of AF [59]. For example, exercise-based cardiac rehabilitation has been associated with 26% lower odds of AF progression and cardiorespiratory rehabilitation programs aimed at increasing peak metabolic equivalents significantly reduce symptoms in AF patients, even after adjusting for weight loss [60, 61]. This highlights the potential for cardiac rehabilitation in this specific group of AF patients, although randomized controlled trials are required to build the evidence required.

Though there are no studies on the effect of treating lung diseases or psychological disorders on dyspnea in AF, it seems reasonable to treat both comorbidities since they are associated with increased dyspnea symptoms in AF patients. Anxiety and depression are common and treating those diseases reduces AF symptoms over time [44]. Symptom burden can further be reduced by improvement of coping skills/acceptation of AF, for example, due to cognitive behavioral therapy.

Optimal pharmacological and non-pharmacological treatment of COPD may improve dyspnea sensation and AF treatment outcomes [28]. Combination therapy with bronchodilators appears to be more effective than monotherapy in reducing dyspnea [62]. Other potential dyspnea-reducing therapies are pulmonary rehabilitation in multi-morbid patients and adjunctive treatment with opioids in stable patients with advanced COPD [63]. Finally, active smoking increases the risk of AF and progression of COPD and smoking cessation should therefore be discussed with all patients [64].

Lastly, when interpreting routine investigations in AF patients such as echocardiography, attention should be paid to uncommon but serious causes of dyspnea such as (chronic thromboembolic) pulmonary hypertension.

## 6 Approach to dyspnea in an AF clinic

In many AF clinics a stepwise approach is used; AF is first treated within a rhythm control approach before the patients without symptom-rhythm correlation and persistent dyspnea are referred to multiple other specialists resulting in a fragmented and burdensome patient journey. Moreover, current guidelines do not specifically focus on the assessment and management of dyspnea in AF patients [33, 65, 66]**(Supplement Table 3)**. In the previous paragraphs we described how a symptom based approach could have a positive effect. Although not yet proven effective, to optimally implement this and to improve dyspnea assessment and management in AF patients, we propose a multidisciplinary integrated care pathway.

### 6,1 Need for a multidisciplinary integrated care approach

**Fig. 2:**
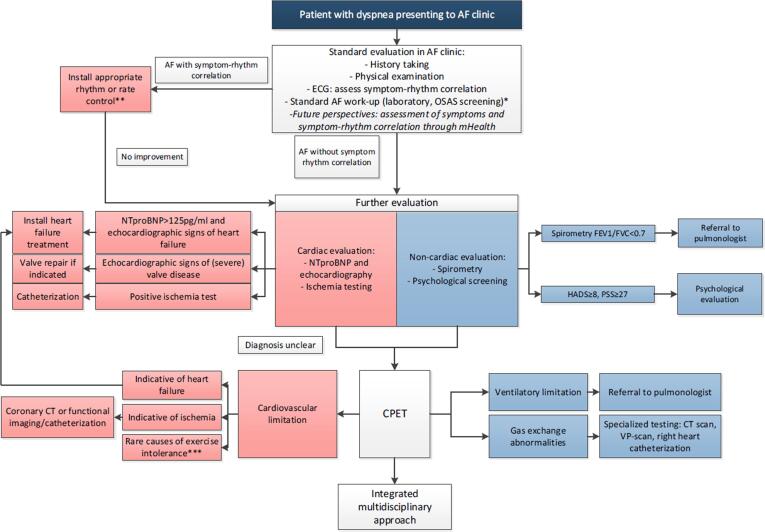
**Example of an integrated care approach.** * Some potential causes of dyspnea are assessed as part of standard AF care (anemia, thyroid disorders). ** NB: also in patients with symptom-rhythm correlation echocardiography should be performed for underlying disease *** Including neuromuscular disease and peripheral vascular disease. Abbreviations: AF = atrial fibrillation, CPET = cardiopulmonary exercise testing, ECG = electrocardiogram, FEV = forced expiratory volume, HADS = Hospital Anxiety and Depression Scale, NTproBNP = N-terminal prohormone B-type natriuretic peptide, OSAS = obstructive sleep apnea syndrome, PSS = Perceived Stress Scale.

The increasing prevalence of multimorbid AF patients necessitates a multidisciplinary and integrated care approach. In such a team multiple specialists work together to assess patients’ needs and to unravel the different components that contribute to the feeling of dyspnea. An example of such an integrated care approach is given in Fig. 2. This integrated care approach can be facilitated by establishing closer collaborations between these specialisms, so that complex cases can be further discussed in multidisciplinary meetings, with specialized nurses (nurse practitioners) acting as staff guided case managers to be transitionally involved in patient care form the hospital level back to general practitioners [67]. Importantly, patients should be actively involved in their care process, receive education and be stimulated to engage in their treatment process. Equally important, lifestyle and risk factor management are also a principal component when it comes to improving dyspnea, for example through involving physiotherapists for cardiopulmonary rehabilitation.

Designing such multidisciplinary teams can be challenging since not all medical specialties (psychologists, physiotherapists, pulmonologists) are readily available in every health care setting. Also, simultaneously assessing patients might carry the risk of overdiagnosis and increase health care costs. However, studies in chronic airway diseases have shown that such a strategy can improve quality of life [68]. Identifying comorbidities can potentially prevent overtreatment of AF which is deemed to fail if dyspnea can be attributed to the presence of other co-morbidities. Additionally, early identification of risk factors and comorbidities might also prevent complications at a later stage.

## 7 Knowledge gaps

Although several large national and international guidelines [33, 65, 66] recommend assessment of symptom burden, a practical guide and standardized care pathway for the management and assessment of dyspnea in AF patients is currently lacking. While the organization of management within a multidisciplinary integrated care approach seems attractive, robust clinical trials assessing the added value of such an approach as well as trials assessing the effects of dyspnea care (improved assessment/management) on patient outcomes are required to finally implement them in clinical practice. Additionally, the effect of cardiopulmonary rehabilitation programs and the relative impact of exercise, risk factor management and patient education remain unclear and require further study.

## 8 Conclusion

Dyspnea is present in up to 66% of AF patients, particularly if AF is accompanied by comorbidities. AF-related as well as comorbidity-related mechanisms can contribute to dyspnea, which may explain the variable symptom-rhythm correlation and incomplete response of dyspnea to rhythm control interventions in dyspneic AF patients. To manage dyspnea in AF patients, we propose an integrated care approach, in which all AF and concomitant comorbidities are targeted within an multidisciplinary team. Further studies are required to evaluate the benefit of such a multidisciplinary integrated care approach to manage dyspnea in AF patients.

## Funding

This research did not receive any specific grant from funding agencies in the public, commercial, or not-for-profit sectors.

## Declaration of Competing Interest

The authors declare that they have no known competing financial interests or personal relationships that could have appeared to influence the work reported in this paper.
