# Supplementary material for: Dyspnea in patients with atrial fibrillation: Mechanisms, assessment and an interdisciplinary and integrated care approach
Source: Int J Cardiol Heart Vasc. 2022 Jul 19;42:101086. doi: 10.1016/j.ijcha.2022.101086 (PMC9304702; doi:10.1016/j.ijcha.2022.101086)
Supplement: Supplementary data 1 [file mmc1.docx]

**Supplementary Material**

**References 51-68**

51. van Os J, Verhagen S, Marsman A, Peeters F, Bak M, Marcelis M, et al. The experience sampling method as an mHealth tool to support self-monitoring, self-insight, and personalized health care in clinical practice. Depress Anxiety. 2017;34(6):481-93.

52. Mehall JR, Kohut RM, Jr., Schneeberger EW, Merrill WH, Wolf RK. Absence of correlation between symptoms and rhythm in "symptomatic" atrial fibrillation. Ann Thorac Surg. 2007;83(6):2118-21.

53. Hermans ANL, Pluymaekers N, Lankveld TAR, van Mourik MJW, Zeemering S, Dinh T, et al. Clinical utility of rhythm control by electrical cardioversion to assess the association between self-reported symptoms and rhythm status in patients with persistent atrial fibrillation. Int J Cardiol Heart Vasc. 2021;36:100870.

54. Björkenheim A, Brandes A, Magnuson A, Chemnitz A, Svedberg L, Edvardsson N, et al. Assessment of Atrial Fibrillation-Specific Symptoms Before and 2 Years After Atrial Fibrillation Ablation: Do Patients and Physicians Differ in Their Perception of Symptom Relief? JACC Clin Electrophysiol. 2017;3(10):1168-76.

55. De With RR, Rienstra M, Smit MD, Weijs B, Zwartkruis VW, Hobbelt AH, et al. Targeted therapy of underlying conditions improves quality of life in patients with persistent atrial fibrillation: results of the RACE 3 study. Europace. 2019;21(4):563-71.

56. Abed HS, Wittert GA, Leong DP, Shirazi MG, Bahrami B, Middeldorp ME, et al. Effect of weight reduction and cardiometabolic risk factor management on symptom burden and severity in patients with atrial fibrillation: a randomized clinical trial. Jama. 2013;310(19):2050-60.

57. Pathak RK, Middeldorp ME, Lau DH, Mehta AB, Mahajan R, Twomey D, et al. Aggressive risk factor reduction study for atrial fibrillation and implications for the outcome of ablation: the ARREST-AF cohort study. J Am Coll Cardiol. 2014;64(21):2222-31.

58. Pathak RK, Middeldorp ME, Meredith M, Mehta AB, Mahajan R, Wong CX, et al. Long-Term Effect of Goal-Directed Weight Management in an Atrial Fibrillation Cohort: A Long-Term Follow-Up Study (LEGACY). J Am Coll Cardiol. 2015;65(20):2159-69.

59. Buckley BJR, Lip GYH, Thijssen DHJ. The counterintuitive role of exercise in the prevention and cause of atrial fibrillation. Am J Physiol Heart Circ Physiol. 2020;319(5):H1051-h8.

60. Buckley BJR, Harrison SL, Fazio-Eynullayeva E, Underhill P, Lane DA, Thijssen DHJ, et al. Association of Exercise-Based Cardiac Rehabilitation with Progression of Paroxysmal to Sustained Atrial Fibrillation. J Clin Med. 2021;10(3).

61. Pathak RK, Elliott A, Middeldorp ME, Meredith M, Mehta AB, Mahajan R, et al. Impact of CARDIOrespiratory FITness on Arrhythmia Recurrence in Obese Individuals With Atrial Fibrillation: The CARDIO-FIT Study. J Am Coll Cardiol. 2015;66(9):985-96.

62. Nici L, Mammen MJ, Charbek E, Alexander PE, Au DH, Boyd CM, et al. Pharmacologic Management of Chronic Obstructive Pulmonary Disease. An Official American Thoracic Society Clinical Practice Guideline. Am J Respir Crit Care Med. 2020;201(9):e56-e69.

63. Spruit MA, Singh SJ, Garvey C, ZuWallack R, Nici L, Rochester C, et al. An official American Thoracic Society/European Respiratory Society statement: key concepts and advances in pulmonary rehabilitation. Am J Respir Crit Care Med. 2013;188(8):e13-64.

64. Zhu W, Yuan P, Shen Y, Wan R, Hong K. Association of smoking with the risk of incident atrial fibrillation: A meta-analysis of prospective studies. Int J Cardiol. 2016;218:259-66.

65. January CT, Wann LS, Alpert JS, Calkins H, Cigarroa JE, Cleveland JC, Jr., et al. 2014 AHA/ACC/HRS guideline for the management of patients with atrial fibrillation: executive summary: a report of the American College of Cardiology/American Heart Association Task Force on practice guidelines and the Heart Rhythm Society. Circulation. 2014;130(23):2071-104.

66. Andrade JG, Aguilar M, Atzema C, Bell A, Cairns JA, Cheung CC, et al. The 2020 Canadian Cardiovascular Society/Canadian Heart Rhythm Society Comprehensive Guidelines for the Management of Atrial Fibrillation. Can J Cardiol. 2020;36(12):1847-948.

67. Leue C, van Schijndel M, Keszthelyi D, van Koeveringe G, Ponds RWHM, Kathol RG, et al. The multi-disciplinary arena of psychosomatic medicine – Time for a transitional network approach. 2020. p. 63-73.

68. McDonald VM, Clark VL, Cordova-Rivera L, Wark PAB, Baines KJ, Gibson PG. Targeting treatable traits in severe asthma: a randomised controlled trial. European Respiratory Journal. 2020;55(3):1901509.

**Supplementary Table 1. Frequently used atrial fibrillation specific symptom scales and questionnaires**

| **Tool** | **Assessment** | **Can only be used during AF episode?** | **Scores** | **Specific symptoms assessed?** | **Advantages** | **Limitations** |
| --- | --- | --- | --- | --- | --- | --- |
| **AF specific scales** | | | | | |  |
| EHRA classification^S9^ | Symptoms and impact on normal daily activities are assessed by physicians by taking the medical history | Yes | EHRA I (no symptoms) to EHRA IV (disabling symptoms) | No, symptoms are summarized into one score | Short, easy to use | Correlate symptoms to presence of AF; possible underestimation of the illness burden in paroxysmal AF and absence of AF episode |
| CCS-SAF scale^S10^ | Combines patient-reported AF-related symptoms, symptom-rhythm temporal correlation and impact of symptoms on function and QoL | Yes | 0-4 | Yes | Short, easy to use | Correlate symptoms to presence of AF; possible underestimation of the illness burden in paroxysmal AF and absence of AF episode |
| **AF specific questionnaires** | | | | | |  |
| University of Toronto AFSS^S11^ | Assessment of AF disease burden including frequency, duration, severity of episodes, symptoms | No | 3-30 | Yes | Symptoms are assessed independently | Designed for research purposes, relatively time-consuming, not designed for AF burden, not to assess dyspnea as a main symptom |
| Symptom-Checklist; Frequency and Severity scale^S12^ | Score based on frequency and severity of symptoms | No | 0-64 and 0-48 for frequency and severity, respectively | Yes | Symptoms are assessed independently | Designed for research purposes, relatively time-consuming, not designed to assess dyspnea as a main symptom |

*NB: The Medical Outcomes Study 36-item Short-Form Health Survey^S13^ is the most used questionnaire in AF research, but does not specifically assess dyspnea as AF-related symptom and is therefore not included in this table.*

*Abbreviations: AF = atrial fibrillation, AFSS = Atrial Fibrillation Severity Scale, CCS-SAF = Canadian Cardiovascular Society Severity of Atrial Fibrillation Scale, EHRA = European Heart Rhythm Association, QoL= Quality of Life. This list is not exhaustive but presents an overview of frequently used scales and questionnaires*

**Supplementary Table 2. Requirements for future assessment methods evaluating dyspnea in atrial fibrillation patients**

| **Requirements for future assessment methods evaluating dyspnea in AF patients** | |
| --- | --- |
| **Assessment** | **Interpretation** |
| - Assessment of symptom-rhythm correlation - Longitudinal assessment with multiple measurements a day for a period of one week up to one month - Measuring both symptom severity and frequency on behalf of dyspnea burden - Measuring multiple dimensions of dyspnea, including sensory-perceptual experience, affective distress, and symptom or disease burden - Easy to apply in clinical practice; time efficient, usable for all patients, easy to interpret | - Including situational/contextual triggers for dyspnea - Including the relationship between health status and dyspnea in AF; i.e. quality of life reduction because of dyspnea - Including negative and positive affectivity - Responsive to intervention |

*Abbreviations: AF = atrial fibrillation*

**Supplementary Table 3. Professional society recommendations on the assessment of dyspnea**

|  | **European Society of Cardiology^S13^** | **American Heart Association/American College of Cardiology/Heart Rhythm Society^S14^** | **Canadian Cardiovascular Society^S15^** |
| --- | --- | --- | --- |
| Recommendations on dyspnea | *‘’Symptom status should be characterized using the EHRA symptom scale and the relation of symptoms (especially if non-specific, such as shortness of breath etc.) to AF should be elucidated because symptoms may also result from undiagnosed or suboptimally managed concomitant cardiovascular risk factors or pathological conditions’’* | *‘’Symptoms should be recorded.’’* | *‘’The presence and nature of AF-related symptoms, their severity, and their effect on QOL should be determined’’* |
| Assessment of dyspnea | Not specified | Not specified | Not specified. Regarding symptoms and QOL*: ‘’A consistent and standardized assessment of the effect of AF on QOL is recommended to evaluate the clinical effect of AF and quantitatively assess the changes in well-being resulting from therapeutic interventions’’* |
| Advised scale | EHRA classification | **-** | CCS-SAF |
| Standardized dyspnea pathway | No | No | No |

*Abbreviations: AF=Atrial fibrillation, CCS-SAF=Canadian Cardiovascular Society Severity of Atrial Fibrillation Scale, EHRA=European Heart Rhythm Association, QOL=Quality of life*

**Supplementary References**

S1. Bin Salih SA, Showlag MS, Al-Qahtani MA, Taha A, Yousuf M, Abdullah M. Clinical characteristics of patients with atrial fibrillation at a tertiary care hospital in the central region of Saudi Arabia. J Family Community Med May 2011;18:80-84.

S2. Blum S, Muff C, Aeschbacher S, et al. Prospective Assessment of Sex-Related Differences in Symptom Status and Health Perception Among Patients With Atrial Fibrillation. J Am Heart Assoc Jun 30 2017;6.

S3. Dhungel S, Laudari S. Clinical Profile of Atrial Fibrillation in a Tertiary Hospital in Central Nepal. JNMA J Nepal Med Assoc Jul-Sep 2017;56:335-340.

S4. Freestone B, Rajaratnam R, Hussain N, Lip GY. Admissions with atrial fibrillation in a multiracial population in Kuala Lumpur, Malaysia. Int J Cardiol Oct 2003;91:233-238.

S5. Guerra F, Brambatti M, Nieuwlaat R, Marcucci M, Dudink E, Crijns H, Matassini MV, Capucci A. Symptomatic atrial fibrillation and risk of cardiovascular events: data from the Euro Heart Survey. Europace Dec 1 2017;19:1922-1929.

S6. Lip GY, Laroche C, Boriani G, et al. Sex-related differences in presentation, treatment, and outcome of patients with atrial fibrillation in Europe: a report from the Euro Observational Research Programme Pilot survey on Atrial Fibrillation. Europace Jan 2015;17:24-31.

S7. Lok NS, Lau CP. Presentation and management of patients admitted with atrial fibrillation: a review of 291 cases in a regional hospital. Int J Cardiol Mar 3 1995;48:271-278.

S8. Schnabel RB, Pecen L, Rzayeva N, Lucerna M, Purmah Y, Ojeda FM, De Caterina R, Kirchhof P. Symptom Burden of Atrial Fibrillation and Its Relation to Interventions and Outcome in Europe. J Am Heart Assoc May 18 2018;7.

S9. Wynn GJ, Todd DM, Webber M, Bonnett L, McShane J, Kirchhof P, Gupta D. The European Heart Rhythm Association symptom classification for atrial fibrillation: validation and improvement through a simple modification. EP Europace 2014;16:965-972.

S10. Dorian P, Cvitkovic SS, Kerr CR, Crystal E, Gillis AM, Guerra PG, Mitchell LB, Roy D, Skanes AC, Wyse DG. A novel, simple scale for assessing the symptom severity of atrial fibrillation at the bedside: the CCS-SAF scale. Can J Cardiol Apr 2006;22:383-386.

S11. Dorian P, Paquette M, Newman D, Green M, Connolly SJ, Talajic M, Roy D. Quality of life improves with treatment in the Canadian Trial of Atrial Fibrillation. Am Heart J Jun 2002;143:984-990.

S12. Bubien RS, Knotts-Dolson SM, Plumb VJ, Kay GN. Effect of radiofrequency catheter ablation on health-related quality of life and activities of daily living in patients with recurrent arrhythmias. Circulation Oct 1 1996;94:1585-1591.

S13. McHorney CA, Ware JE, Jr., Raczek AE. The MOS 36-Item Short-Form Health Survey (SF-36): II. Psychometric and clinical tests of validity in measuring physical and mental health constructs. Med Care Mar 1993;31:247-263.

S14. Hindricks G, Potpara T, Dagres N, et al. 2020 ESC Guidelines for the diagnosis and management of atrial fibrillation developed in collaboration with the European Association of Cardio-Thoracic Surgery (EACTS). Eur Heart J Aug 29 2020.

S15. January CT, Wann LS, Alpert JS, et al. 2014 AHA/ACC/HRS guideline for the management of patients with atrial fibrillation: executive summary: a report of the American College of Cardiology/American Heart Association Task Force on practice guidelines and the Heart Rhythm Society. Circulation Dec 2 2014;130:2071-2104.

S16. Andrade JG, Aguilar M, Atzema C, et al. The 2020 Canadian Cardiovascular Society/Canadian Heart Rhythm Society Comprehensive Guidelines for the Management of Atrial Fibrillation. Can J Cardiol Dec 2020;36:1847-1948.
